# Supplementary material for: The mechanical microenvironment regulates ovarian cancer cell morphology, migration, and spheroid disaggregation
Source: Sci Rep. 2018 May 8;8:7228. doi: 10.1038/s41598-018-25589-0 (PMC5940803; doi:10.1038/s41598-018-25589-0)
Supplement: Supplementary file 4 — Supplementary Information [file 41598_2018_25589_MOESM4_ESM.pdf]

The mechanical microenvironment regulates ovarian cancer cell morphology, migration, and spheroid disaggregation

Andrew J. McKenzie, Stephanie R. Hicks, Kathryn V. Svec, Hannah Naughton, Zöe L. Edmunds, and Alan K. Howe*

Supplementary Information

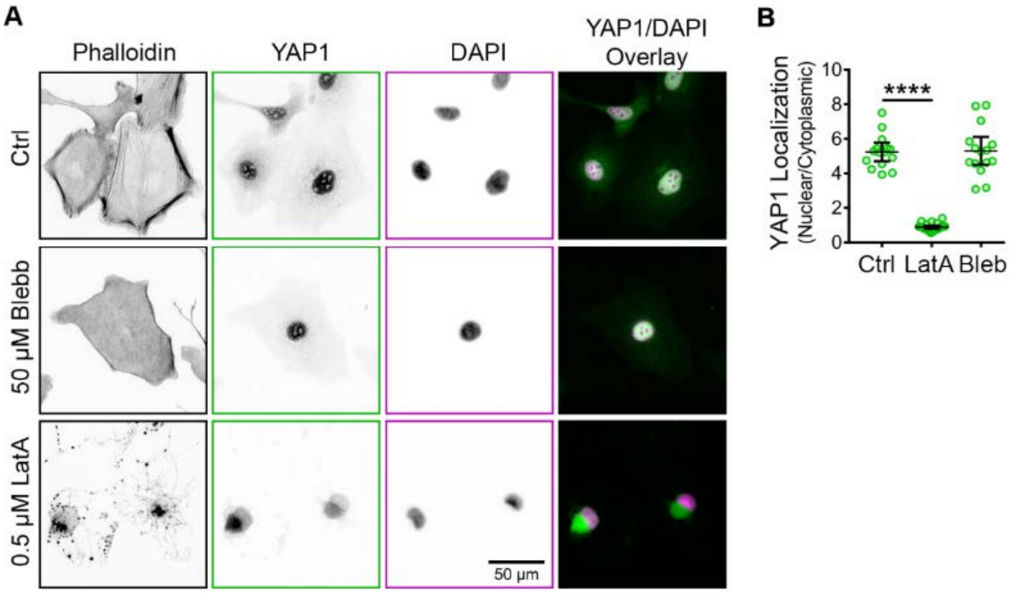

Supplemental Figure 1. Nuclear translocation of YAP1 is independent of actomyosin contractility but dependent on F-actin cytoskeleton integrity. (A) YAP1 localization shown for representative cells exposed to X µM DMSO (ctrl, top row), 50 µM blebbistatin (Blebb), or 0.5 µM latrunculin A (Lat A) for 4 hours before being fixed and stained to visualize F-actin, YAP1, and nuclei. (B) Quantification of YAP1 localization given as a ratio of YAP1 signal in the nucleus to YAP1 signal in the cytoplasm for cells in each treatment condition. The graph depicts all measure values (colored symbols) and the mean values (± s.d.; n = 15 for DMSO and latrunculin A and 21 for blebbistatin; *p* = < 0.0001 using the Mann-Whitney test).

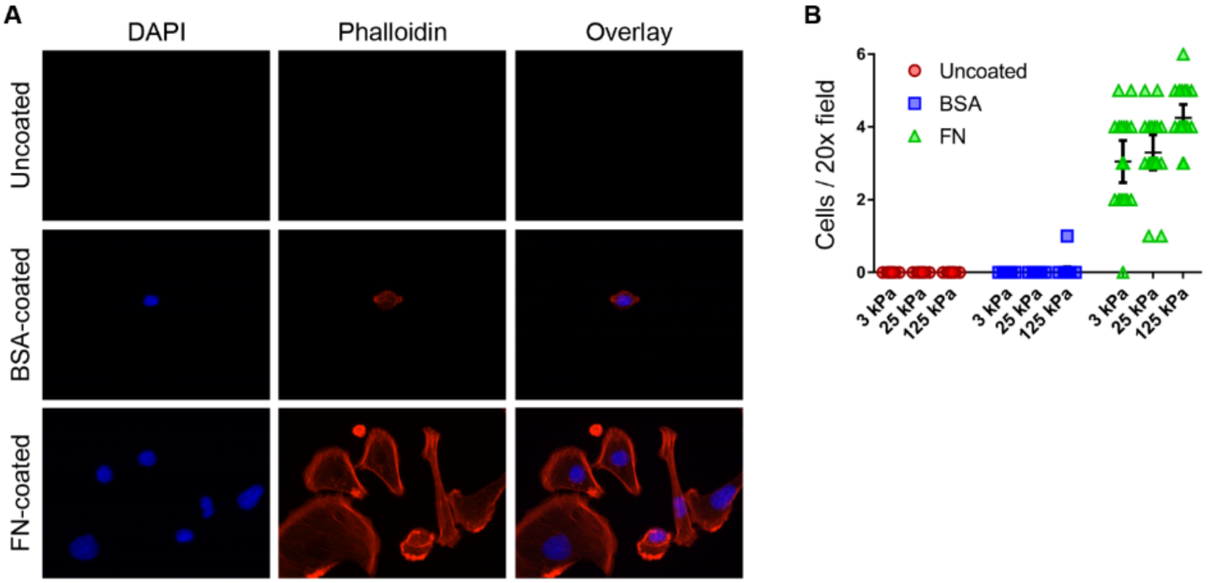

Supplemental Figure 2. Cell adhesion and morphology on hydrogels is dependent on fibronectin. (A) SKOV-3 cells plated were seeded at 5000 cells / cm^2 on 125 kPa hydrogels that were uncoated, coated with 20 µg/ml BSA, or 20 µg/ml FN, incubated under standard tissue culture conditions overnight. Hydrogels were processed for fluorescence staining (as described in *Methods*; briefly, two washes in PBS followed by formaldehyde fixation and detergent permeabilization), then stained with DAPI and phalloidin to visualize nuclei and F-actin respectively. (B) Uncoated, BSA-coated, or FN-coated hydrogels of the indicated Young's modulus (3, 25, or 125 kPa) were seeded with SKOV-3 cells, then fixed and stained as in (A). Twenty 20x microscope fields were imaged and the number of cells per field were counted. Of note, the single cell on the BSA-coated gel shown in panel (A) was the only cell found in all of the uncoated or BSA-coated fields combined (B).

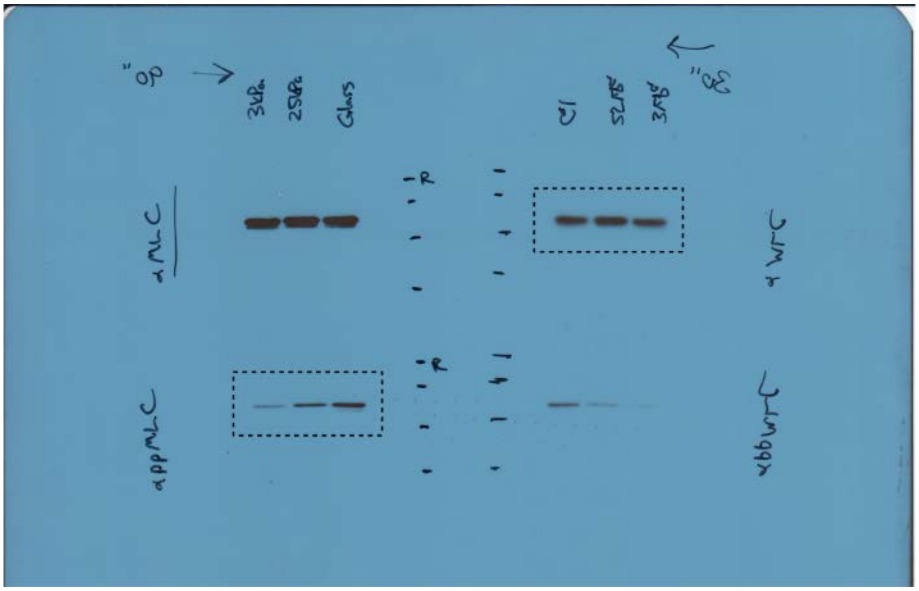

Supplemental Figure 3. Uncropped immunoblot for panels in Figure 3. Note that the left & right halves of the blot are mirror images; the film was flipped horizontally to capture 30 & 90 sec exposures of the blotted membranes.
